# ATP-induced cell death: a novel hypothesis for osteoporosis

**DOI:** 10.3389/fcell.2023.1324213

**Published:** 2023-12-14

**Authors:** Wei Wang, Haolong Zhang, Doblin Sandai, Rui Zhao, Jinxia Bai, Yanfei Wang, Yong Wang, Zhongwen Zhang, Hao-Ling Zhang, Zhi-Jing Song

**Affiliations:** ^1^ College of Acupuncture-Moxibustion and Tuina, Gansu University of Chinese Medicine, Lanzhou, Gansu, China; ^2^ Department of Biomedical Sciences, Advanced Medical and Dental Institute, Universiti Sains Malaysia, Kepala Batas, Penang, Malaysia; ^3^ Clinical College of Chinese Medicine, Gansu University of Chinese Medicine, Lanzhou, Gansu, China; ^4^ Pathology Center, Gansu University of Chinese Medicine, Lanzhou, Gansu, China; ^5^ School of Public Health, Gansu University of Chinese Medicine, Lanzhou, Gansu, China

**Keywords:** ATP-induced cell death, osteoporosis, bone marrow-derived mesenchymal stem cells, osteoblasts, osteoclasts, complications, apoptosis, autophagy

## Abstract

ATP-induced cell death has emerged as a captivating realm of inquiry with profound ramifications in the context of osteoporosis. This study unveils a paradigm-shifting hypothesis that illuminates the prospective involvement of ATP-induced cellular demise in the etiology of osteoporosis. Initially, we explicate the morphological attributes of ATP-induced cell death and delve into the intricacies of the molecular machinery and regulatory networks governing ATP homeostasis and ATP-induced cell death. Subsequently, our focus pivots towards the multifaceted interplay between ATP-induced cellular demise and pivotal cellular protagonists, such as bone marrow-derived mesenchymal stem cells, osteoblasts, and osteoclasts, accentuating their potential contributions to secondary osteoporosis phenotypes, encompassing diabetic osteoporosis, glucocorticoid-induced osteoporosis, and postmenopausal osteoporosis. Furthermore, we probe the captivating interplay between ATP-induced cellular demise and alternative modalities of cellular demise, encompassing apoptosis, autophagy, and necroptosis. Through an all-encompassing inquiry into the intricate nexus connecting ATP-induced cellular demise and osteoporosis, our primary goal is to deepen our comprehension of the underlying mechanisms propelling this malady and establish a theoretical bedrock to underpin the development of pioneering therapeutic strategies.

## 1 Introduction

As the worldwide population continues to age, the prevention and management of osteoporosis have assumed paramount significance (Clynes et al., 2020). Osteoporosis is a common skeletal condition that is primarily characterized by decreased bone mineral density, cortical thinning, and increased fracture risk (Wang et al., 2023a; Cavati et al., 2023).

Cellular apoptosis, autophagy, and necroptosis play pivotal roles in the pathogenesis of various bone diseases. It is widely believed that the dysplasia of osteoblasts and osteoclasts is a factor leading to osteoporosis (Xiao et al., 2023). Estrogen depletion, inflammatory responses, and immune dysregulation [including tumor necrosis factor-alpha (TNF-α) and interleukin-1 beta (IL-1β)] in conjunction with disturbances in calcium ion homeostasis can independently or synergistically trigger apoptotic, autophagic, and necroptotic processes in bone cells, culminating in the onset of osteoporosis (Wang et al., 2023b; Cui et al., 2023; Feng et al., 2023; Jeong et al., 2023; Puts et al., 2023; Xiao et al., 2023; Zhu et al., 2023). Furthermore, aberrant activities of osteoclasts and multinucleated macrophages result in the release of acidifying substances and bone resorption-related proteases, contributing to bone degradation and subsequent osteoporosis (Bao et al., 2023) Importantly, cell death pathways are also implicated in secondary osteoporosis, encompassing conditions such as diabetic osteoporosis, glucocorticoid-induced osteoporosis, and postmenopausal osteoporosis.

ATP (adenosine triphosphate), a multifunctional signaling molecule both intra- and extracellularly, plays a crucial part in the metabolism of cellular energy and is deeply entangled in the control of cell function and disease development. While previous investigations primarily focused on the release and impact of ATP in neuronal activity and neurotransmission, recent findings have uncovered its ability to induce cell death, termed ATP-induced cell death, when extracellular ATP levels are elevated (Placido et al., 2006). Moreover, A growing body of research indicates that ATP release may cause bone cells to apoptose and launch an inflammatory response, potentially impacting the development of osteoporosis (Kvist et al., 2014; Walsh et al., 2018).

In this study, we elucidate the impact of ATP-induced cell death on the etiology of primary and secondary osteoporosis, while concurrently investigating the intricate interplay between ATP-induced cell death and the constituents of apoptosis, autophagy, and necrosis. This thorough analysis gives a novel conceptual framework and offers a strong basis for the creation of therapeutic approaches meant to modulate ATP-induced cell death in order to lessen or cure primary and secondary osteoporosis.

## 2 Morphological characteristics of ATP-induced cell death

ATP-induced cell death typically manifests as apoptosis, while a portion of it manifests as an alternative form of cell death known as necrosis (Schulze-Lohoff et al., 1998; Tamajusuku et al., 2010; Souza et al., 2012). ATP-induced cell death exhibits distinct morphological characteristics, including cellular shrinkage and loss of connectivity. Necrotic cells undergo a reduction in size and detachment from neighboring cells. Moreover, cytoplasmic density increases, and Mitochondrial membrane potential is damaged, leading to alterations in mitochondrial permeability and the release of cytochrome C into the cytoplasm. Nucleolar concentration and fragmentation occur as the nuclear membrane and nucleolus disintegrate during necrotic cell death. As a result of the subsequent DNA (deoxyribonucleic acid)degradation, fragments with a size of 180–200 base pairs arise. degradation ensues, resulting in the formation of fragments approximately 180–200 base pairs in size, while the membrane forms vesicular structures and exposes phosphatidylserine on its surface. Notably, apoptotic bodies are generated, devoid of cellular contents, thereby eluding an inflammatory response. These apoptotic bodies are swiftly engulfed by neighboring phagocytes (Häcker, 2000). Furthermore, cell necrosis is accompanied by increased membrane permeability, causing cellular swelling, organelle deformation, or enlargement. While early stages show no significant nuclear morphological changes, eventual cell rupture ensues, releasing cellular contents and frequently eliciting an inflammatory response. Healing processes following cell necrosis are often accompanied by tissue and organ fibrosis, leading to scar formation (Krysko et al., 2008). ATP-induced cell death can manifest as either apoptosis or cell necrosis, contingent upon various factors, such as cell type, ATP concentration, duration of ATP action, intracellular and extracellular environmental conditions, and the cumulative effects of these factors.

## 3 Mechanism and regulation of ATP homeostasis and ATP-induced cell death

**FIGURE 1:**
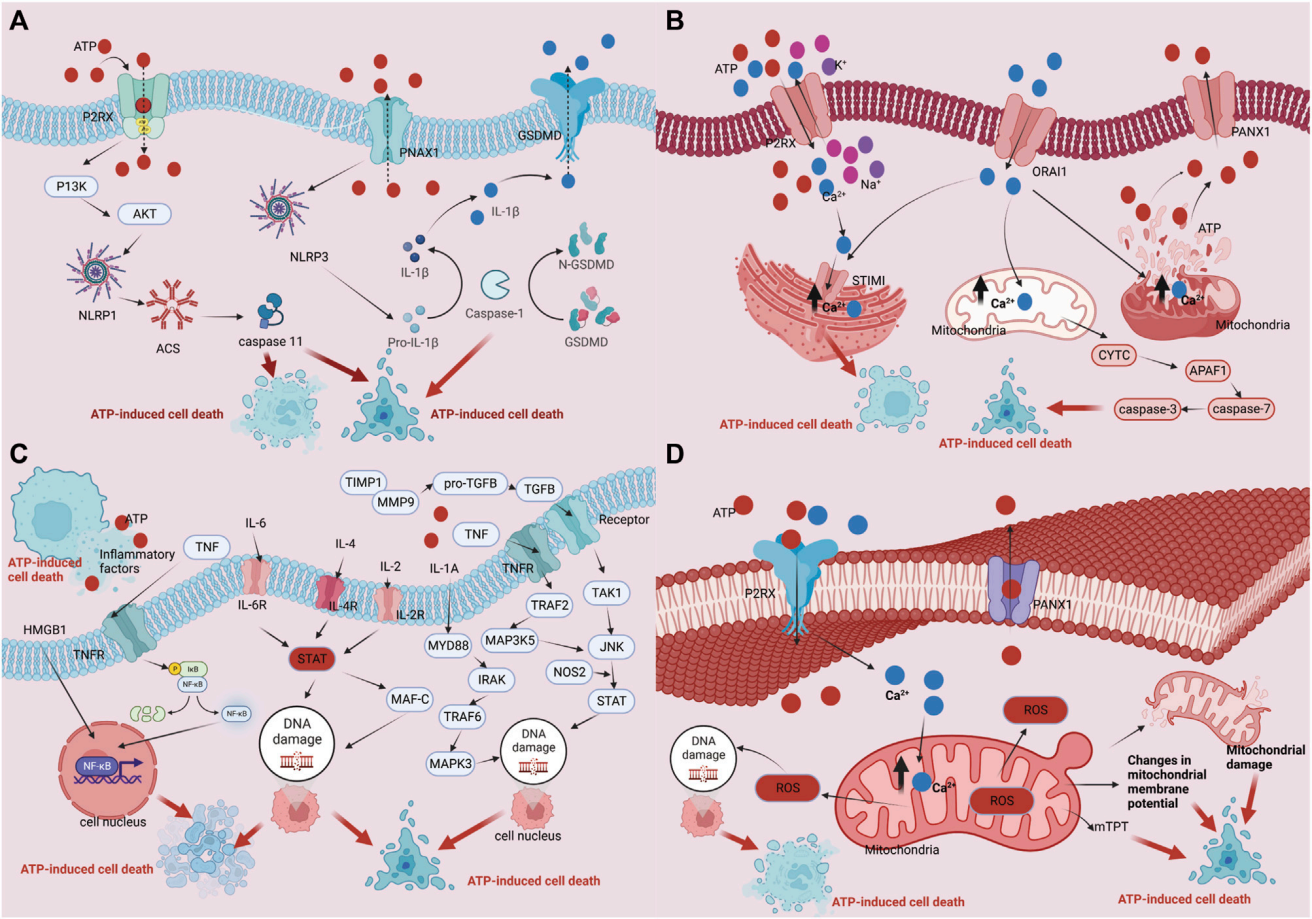
Regulation mechanism of ATP homeostasis and ATP-induced cell death: **(A)** P2 receptor activation pathway; **(B)** Ca^2+^ pathway induces cell death pathways; **(C)** ATP cell death releases immune inflammatory factors as well as immune pathways that induce cell death; **(D)** The depletion of mitochondrial membrane potential, disruption of mitochondrial integrity, generation of reactive oxygen species (ROS), and alterations in mitochondrial membrane permeability collectively culminate in cellular demise.

ATP fulfills crucial biological functions within cells, encompassing energy transfer, signal transduction, and cellular metabolism. Intracellular ATP levels are upheld by the delicate equilibrium between intracellular synthesis and consumption, known as ATP homeostasis. However, under external stimulation or internal injury, ATP homeostasis may be disrupted, resulting in an increase in extracellular ATP levels and the release of intracellular ATP. The result of this action is ATP-induced cell death. There are four main mechanisms and regulatory pathways that might cause ATP-induced cell death. Firstly, ATP can engage with extracellular purinergic 2 receptors (P2Rs), particularly P2X7 receptors, concurrently activating associated receptors such as NLRP1 (NOD-like receptor heat protein domain-associated protein 1) and NLRP3 (NOD-like receptor heat protein domain-associated protein 3). This activation initiates apoptotic signals, including caspase-1, caspase-3, and caspase-11, and involves necrotic proteins such as GSDME (Gasdermin-E) and GSDMD (Gasdermin-D). The culmination of these activations ultimately sets in motion the cell death process (Perregaux and Gabel, 1998; Ono et al., 2003; Piccini et al., 2008; Shiratori et al., 2010; Shoji et al., 2014; Toki et al., 2015; Amati et al., 2017a; Amati et al., 2017b; He et al., 2017). Secondly, The ability of ATP to bind to ion channels on the cell membrane helps to control the balance of ions within and outside of the cell. For instance, activation of P2R (purinergic 2 receptor), particularly the P2X7R, causes ion channels to open, increasing the concentration (including within the Golgi apparatus and mitochondria) of Ca^2+^ inside of cells. Consequently, nuclear DNA damage occurs, leading to cell death (Kim et al., 2005; Sehgal et al., 2017). Furthermore, ATP can induce a change in the loss of mitochondrial membrane potential (due to K^+^/Na^+^ imbalance on the cell membrane), disruption of the mitochondrial respiratory chain, production of reactive oxygen species (ROS), and altered mitochondrial membrane permeability, ultimately triggering cell death (Souza et al., 2012; Xue et al., 2016). Ultimately, it participates in the initiation of immune inflammation, activation of cell death, and ATP-induced cell death, resulting in the release of inflammatory mediators such as IL-1β, IL-18 (interleukin-18), TNF-α, IL-2 (interleukin-2), IL-4 (interleukin-4), IL-6 (interleukin-6), IL-a (interleukin-A), CCL5 (chemokine (C-X-C motif) ligand 5), and CXCL2 (chemokine (C-X-C motif) ligand 2), ultimately culminating in cell death. (refer to Figure 1) (Zheng et al., 1991; Di Virgilio, 1995; Li et al., 2021).

## 4 Potential metabolic role of ATP-induced cell death in osteoporosis

Osteoporosis, a growing medical and socioeconomic threat, is characterized by a general decline in bone density, strength, and microstructure that increases the risk of fragility fractures. Although prior research mainly connected osteoporosis to changes in calcium metabolism, it is significant that studies have found a loss in the density of bone minerals beginning at the age of 30 and continuing into old life. Additionally, growing clinical data strongly links osteoporosis to ATP-induced cell death, particularly in postmenopausal women. The pathophysiology of osteoporosis assumes a supervisory role for ATP-induced cell death. The constant remodeling of bone, a metabolically active tissue, which is regulated by osteoclasts’ resorption of bone and osteoblasts’ synthesis, keeps the skeleton in balance. Surprisingly, ATP-induced cell death has been found to promote osteoclastogenesis and trigger osteocyte apoptosis through the release of RANKL, a cytokine that increases the formation of osteoclasts. This emphasizes the important role that ATP-induced cell death plays in the production of osteoclasts.

### 4.1 ATP induction of bone mineral mesenchymal stem cell death

The research of bone marrow mesenchymal stem cells (BMSCs) has received a great deal of attention recently due to developments in the field of bone tissue repair and regeneration. Low immunogenicity, extensive accessibility, and enormous potential for varied differentiation are the distinguishing characteristics of BMSCs. They either directly differentiate into injured tissues and organs to help with tissue and organ repair or produce exosomes, growth factors, and cytokines. Notably, the role played by BMSCs in controlling ATP-induced cell death, which has received considerable attention. ATP, for example, stimulates the PI3K/Akt/(Protein Kinase B), ERK (extracellular regulated protein kinases), and JNK(c-Jun N-terminal kinase) signaling pathways via P2X7R binding to BMSCs, triggering death and hindering osteocyte differentiation (Noronha-Matos et al., 2014). The activation of the P2X7-Rho kinase pathway triggers a reversible reorganization of the cytoskeleton, which enhances osteogenic differentiation and mineralization of BMSCs. This offers a new potential therapy for the treatment of common postmenopausal bone loss (Li et al., 2015). Additionally, ATP induces the expression of genes for osteogenic markers, alkaline phosphatase activity, and bone mineralization upon P2X7R activation, increasing bone trabecular volume and number (Xu et al., 2019; Mittermayr et al., 2021). Recent studies have shown that shockwaves support intracellular ATP release, P2X7R activation, and downstream signaling activities, eventually boosting p38MAPK-mediated osteogenic activation in BMSCs and improving bone healing (Zeng et al., 2019a; Fan et al., 2020). Moreover, ATP regulates the oxidative stress and mitochondrial function of BMSCs, further affecting their capacity for osteogenic differentiation (Zeng et al., 2019a).

**FIGURE 2:**
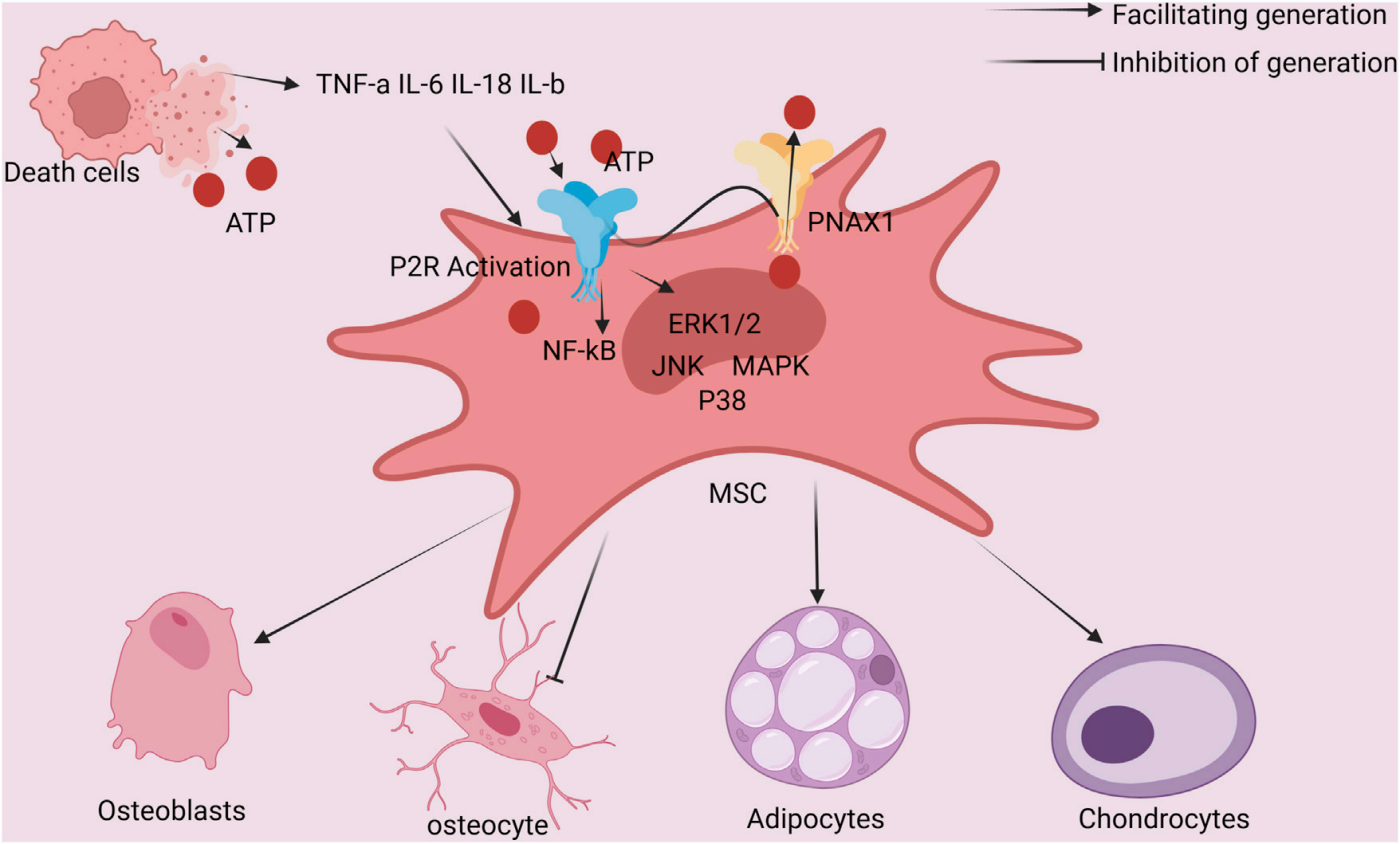
Schematic diagram of the interaction between ATP-induced cell death and bone Mesenchymal stem cells of the bone marrow: ATP activation of cells shows P2R, cell autosecretion of IL-1 and other factors, whereas ATP induction of cell death releases IL-18 and other factors that act on mesenchymal stem cells of the bone marrow. Additionally, P2R-positive cells that are stimulated by ATP also activate the P38 MAPK/JNK/ERK1/2/NF-kB signaling pathway, which encourages mesenchymal stem cells in the bone marrow to develop into OBs.

Mesenchymal stem cells (MSCs) give rise to osteoblast differentiation. The release of endogenous ATP in this phase triggers the activation of P2X7R, which then encourages the differentiation of MSCs into osteoblasts. Osteoblast activity is inhibited by ATP derivatives and pyrophosphate (PPi), which are both created when extracellular ATP is broken down. On the other hand, endogenous ATP has a pro-osteogenic effect whether it is present at basal levels or is produced mechanically. Prostaglandin E2 (PGE2) synthesis/release, lysophosphatidic acid (LPA) synthesis and secretion, and serine/threonine kinase (ERK12) activation are just a few of the downstream events that are brought on by P2X7R signaling cascades and contribute to the enhancement of osteoblast differentiation and the promotion of bone tissue formation. By causing the release of ATP in response to mechanical stimulation, the P2X7R-mediated osteogenesis may be amplified. Osteoblast differentiation and matrix mineralization are fostered by brief P2X7R agonist activation. Apoptosis can be induced and anti-osteogenic effects can be seen when stimulation is prolonged because it can lead to extracellular ATP buildup (Figure 2) (Agrawal and Gartland, 2015; Noronha-Matos and Correia-de-Sá, 2016; Ma et al., 2022; Huang et al., 2023).

*In vitro* bone nodule mineralization has been shown to be inhibited by activation of the purinergic 2X1 and 2X3 receptors (P2X1 and P2X3R, respectively) (Boeynaems, 2016). Purinergic 2Y receptors, which are expressed functionally by mesenchymal stem cells (MSCs), are subject to expression level modulation during osteoblast or adipocyte differentiation.

The production of colony-forming units of alkaline phosphatase (CFU-ALP) in adherent bone marrow-derived MSCs is dramatically increased when P2Y13R (purinergic 2Y13 receptor) is stimulated (Li et al., 2016). Purinergic 2Y2 receptor activation was found to decrease osteogenesis and enhance lipogenic differentiation of BMSCs, with the ERK1/2 signaling pathway mediating the initial phases of this process (Du et al., 2018). TNF- inhibits the osteogenic differentiation of MSCs, impairing bone production and being a key player in the pathophysiology of postmenopausal osteoporosis. The P2Y2R gene is highly upregulated by TNF- in MSCs as they differentiate into osteoblasts. The osteogenic development of MSCs (mesenchymal stem cells), whether through overexpression or silencing, is significantly impacted by manipulation of P2Y2R expression. When the P2Y2R is silenced, TNF- has less of an inhibitory effect on MSC osteoblast differentiation and much less of an impact on MSC proliferation. TNF- mainly controls the ERK and JNK signaling pathways to regulate the expression of the P2Y2R (Yuan et al., 2018). These studies propose scientific hypotheses for the treatment of patients with osteoporosis.

As observed in ATP-induced cell death, TNF- activates nuclear factor-B(NF-κB) and degrades -catenin. Quercetin stimulates the proliferation of rat BMSCs and alleviates osteoporosis. *In vivo*, quercetin improves bone mineral density (BMD) and enhances bone biomechanical properties in rat models of osteoporosis after menopause. *In vitro*, quercetin significantly inhibits TNF--induced NF-κB activation and -catenin degradation while also promoting the proliferation and osteogenic differentiation of BMSCs (Chen et al., 2021). The impairment of mitochondria is a prominent characteristic of ATP-induced cell death and significantly affects osteoblast differentiation, mineralization, and BMSC survival. A study revealed that the downregulation of Apelin expression is observed in rats with osteoporosis induced by oophorectomy. However, administration of Apelin-13 triggers mitochondrial autophagy in BMSCs, attenuates oxidative stress, and rejuvenates osteogenic activity via AMPK-alpha phosphorylation (Ni et al., 2020). Moreover, Apelin-13 administration rescues bone density and microstructure, rejuvenates mitochondrial autophagy, and augments osteogenic activity in ovariectomized rats (Souza et al., 2012). These findings further accentuate the valuable insights offered by ATP-induced cell death in the management of osteoporosis patients.

### 4.2 Dual role of ATP-induced cell death in osteoblasts

Sustaining bone strength requires a delicate balance between the activities of osteoblasts and osteoclasts, as bone remodeling continues in an unending cycle. Osteoblasts play a crucial role in bone remodeling by overseeing the formation, mineralization, and structure of bone cells. Research has shown the presence of P2X7 purinergic receptors in differentiated human osteoblasts. In experiments using MC3T3-E1 cells, a combination treatment of Wnt3a and the P2X7 agonist 2′ (3′)-O-(4-benzoylbenzoyl) adenosine 5′-triphosphate (BzATP) resulted in a prolonged localization of beta-catenin in the nucleus compared to Wnt3a treatment alone. Furthermore, the addition of BzATP enhanced the transcriptional activity of beta-catenin induced by WNT3A. An ATP concentration of 1 mM amplified the transcriptional activity of beta-catenin induced by WNT3A, whereas lower concentrations (10 M) of ATP, adenosine 5′-diphosphate (ADP), or uridine 5′-triphosphate (UTP) failed to elicit a response (Grol et al., 2016). Furthermore, the administration of external nucleotides has displayed the ability to induce the expression of markers that identify osteoblasts and improve the process of mineralization in cultures of cranial cells from rats through the activation of the P2X7R. This receptor is associated with the production of potent lipid mediators, specifically referred to as LPA and PGE2. The promotional impact of P2X7R activation on osteogenesis can be negated by antagonists of the LPA receptor or inhibitors of cyclooxygenase (COX). As a result, the P2X7R is implicated in the production of LPA and COX metabolites, thereby facilitating osteogenesis (Panupinthu et al., 2008). Activation of the P2X7R can increase the expression of markers that identify osteoblasts, heighten the process of mineralization in cranial cells from rats, and enhance the functionality of osteoblasts through intracellular mechanisms. P2X7 signaling strengthens the well-known Wnt pathway by inhibiting GSK3 (Glycogen synthase kinase-3), ultimately prolonging and enhancing Wnt-catenin signaling. The interplay between the P2X7 and Wnt-catenin pathways potentially regulates the responses of osteoblasts to mechanical loading.

The underlying processes and pharmacological substances involved in controlling osteoblast cell death produced by ATP have been the subject of several studies. The inflammatory cytokines IL-1, TNF-, and RANKL are inhibited by LL-37 through the P2X7R and MAPK (mitogen-activated protein kinase) signaling pathways. This decreases the amount of osteogenesis that is inhibited by LPS (lipopolysaccharides) (Yu et al., 2018). Physical therapy has demonstrated promise in boosting bone formation by regulating the P2X7R in addition to medication. For instance, bone growth in OBs is promoted by low-intensity pulsed ultrasound (LIPUS) physiotherapy via the P2X7R, which increases extracellular ATP release. Another research found that the P2YR repertoire is activated by ADP, ATP, UTP, and UDP (Uridine diphospate), which increases osteoblast proliferation and intracellular Ca^2+^ levels. OBs express several P2YR subtypes, including P2Y1R (purinergic 2Y1 receptor), P2Y2, P2Y4, P2Y6, P2Y11 (purinergic 2Y11), P2Y12 (purinergic 2Y12), and P2Y13. By releasing purines like ATP that predominantly stimulate P2Y1R, LIPUS therapy causes osteoblast generation (Alvarenga et al., 2010). It has also been shown that P2Y1R is involved in the proliferation of osteoblasts and the ADP-mediated rise of intracellular Ca^2+^ levels. In contrast to P2X1, P2X2R, and P2X3R, P2X4 (purinergic 2X4), P2X5R (purinergic 2X5 receptor), P2X6 (purinergic 2X6), and P2X7Rs were shown to be expressed in MG-63 cells. A rise in intracellular Ca^2+^ concentration was induced in FurA2-loaded cells by both ATP and UTP. Additionally, a number of Ca^2+^ inhibitors that target active protein kinases failed to affect ATP-induced DNA synthesis, indicating that an increase in intracellular Ca^2+^ levels is not required for ATP-induced DNA synthesis. According to these results, ATP increases MAPK activity in a Ca^2+^-independent way and synergistically boosts kinase activity brought on by platelet-derived growth factor or insulin-like growth factor I (Nakamura et al., 2000; Bonora et al., 2012). On the basis of these results, it may be predicted that ATP-induced osteoblast cell death might be a way to limit bone resorption, potentially offering an alternate method for the treatment of bone production diseases.

Recent inquiries within cellular biology have elucidated that ATP can instigate cell demise via a plethora of mechanisms, which intriguingly seem to concomitantly enhance osteoblast differentiation. When is a cell undergoing cell death? What are the conditions into osteoblast differentiation? This is a noteworthy topic. Cells may experience death in response to energy depletion or severe damage, such asoxidative stress and DNA damage in ATP-induced cell death or processes (Payne et al., 1995; Barzilai and Yamamoto, 2004; Wang et al., 2006; Lans et al., 2012; Roos and Kaina, 2013; Ma et al., 2014). Conversely, osteoblast differentiation, critical to osseous genesis and recuperation, may be augmented by the activation of signaling conduits, notably by osteotrophic factors such as Wnt3a and BMP2. These agents expedite differentiation through the Akt pathway, augmenting mitochondrial oxidative phosphorylation, signifying a symbiotic nexus between cell death and differentiation processes (Smith and Eliseev, 2021).

### 4.3 ATP induced cell death with osteoclasts

When compared to healthy people, osteoporosis sufferers exhibit upregulated P2X7R expression, which is also accompanied by elevated calcium ion levels. This observation suggests that ATP-induced cell death plays a role in the development of osteoporosis (Kvist et al., 2017). Furthermore, the elevated expression of P2X7R is linked to the downstream activation of calcium/calcineurin/nuclear factor signaling mediated by activated T cell 1 (NFATc1) and increased expression of proteins related to autophagy during the differentiation of osteoclasts (Ma et al., 2022). The activation of P2X7R regulates osteoclast formation and activity through the control of NF-B, while the activation of the P2Y6 nucleotide receptor promotes the survival of osteoclasts (Korcok et al., 2004; Korcok et al., 2005). The fusion of mononuclear precursor cells is a crucial process in osteoclastogenesis, leading to the formation of mature multinucleated osteoclasts. P2X7R facilitates osteoclast fusion by increasing the levels of extracellular adenosine (Pellegatti et al., 2011). P2Y1 and P2Y2R are also expressed in osteoclasts, but their functional mechanisms have not been reported. Upon direct activation of osteoclasts, ATP primarily impacts bone resorption through the P2R subtype, rather than the P2Y2 subtype. Locally released ATP acts as a response to acute trauma or short-term physical stress within the bone microenvironment, influencing the bone remodeling process by interacting with P2R expressed by osteoclasts and OBs (Bowler et al., 1998).

## 5 Potential relationship between ATP-induced cell death and osteoporosis complications

By involving calcium ion buildup and P2R activation more heavily than apoptosis, autophagy, necrosis, and pyroptosis, ATP-induced cell death sets itself apart from these other processes. Notably, ATP-induced cell death and excessive extracellular ATP are tightly related. ATP overload, P2R activation, calcium ion buildup, mitochondrial dysfunction, and peroxide accumulation all interact to cause bone degeneration, which leads to bone diseases.

### 5.1 ATP-induced cell death and diabetic osteoporosis

Diabetic osteoporosis, a common complication of diabetes, is characterized by reduced bone density and strength, making the bones more susceptible to fractures. The development of diabetic osteoporosis involves complex interactions among various factors. Notably, high blood sugar levels (hyperglycemia) play a significant role in contributing to diabetic osteoporosis. Prolonged hyperglycemia negatively affects the formation and absorption of bone tissue, impairs the function of osteoblasts (cells responsible for bone formation), and stimulates the activity of osteoclasts (cells responsible for bone resorption), ultimately leading to the development of osteoporosis. A wealth of evidence supports the idea that elevated glucose levels have an inhibitory effect on the proliferation of MC3T3-E1 cells, a type of bone cell. This inhibition is accompanied by a decrease in the expression of P2X7, a protein involved in cell signaling, resulting in reduced levels of calcification and decreased expression of ALP (alkaline phosphatase) and osteocalcin (Ocn) in MC3T3-E1 cells. On the other hand, overexpression of P2X7 under high glucose conditions enhances calcification and cell proliferation, and increases the expression of ALP and Ocn in MC3T3-E1 cells. Inhibiting P2X7 under high glucose conditions leads to a decrease in the expression of ALP and Ocn in MC3T3-E1 cells (Yang et al., 2019). These findings provide further insights into the crucial role of P2X7 in diabetic osteoporosis, particularly in the context of hyperglycemia.

Additionally, studies have shown that emodin can inhibit the expression of P2X2/3 and have a positive impact on diabetes, giving rise to concrete proof that osteoporosis can be prevented (Dong et al., 2020). A substance with a wide range of natural functions, emodin, has restorative or reversive effects on diabetic osteoporosis by acting on various targets related to diabetes. Studies have also shown that coumarin and its derivatives can upregulate P2X3 63’s expression (Pan et al., 2022). Pain perception is influenced by the P2X3R, a non-selective ligand-gated cation channel mostly found in sympathetic and visual neurons as well as the lone nucleus. Pain associated with diabetic osteoporosis can be alleviated by reducing expression or desensitizing, whereas upregulation or increased activity of the P2X3R may exacerbate pain perception. Notably, it has been demonstrated that hoserine inhibits the P2X3R’s expression in diabetic rats with stellate sympathetic ganglion (SG). In comparison to the glycemic group, immunohistochemical and Western blot studies showed a significant decrease in P2X3 expression in the SG of hops-treated diabetes rats, indicating that hop therapy may be able to counteract this upregulation (Wu et al., 2020). These results support the idea that glycemic fracture can be treated with ATP-induced cell death.

### 5.2 ATP-induced cell death and glucocorticoid-induced osteoporosis

Fracture brought on by these drugs is referred to as glucocorticoid-induced osteopolisis, which develops from long-term or excessive use of these hormones. In medical settings, glycocorticoids are frequently used to treat a variety of aggressive and immune-related disorders. However, their long-term or high-dose management can have a negative effect on bone health, weakening bone density and strength and increasing the risk of injuries. There are many different facets to the pathogenesis of glucocorticoid-induced fracture. First off, glucocorticoids interfere with bone cells’ regular processes, prevent the formation and operation of osteoblasts, and encourage osteoclast activity, which causes bone tissue to become imbalanced and deteriorate. Second, glucocorticoids can affect calcium intake, usage, and excretion within bone tissue by interfering with calcium metabolism and hormonal regulation, endangering bone health even more.

A research showed that the lack of P2X7R led to increased bone loss using a mouse model of estrogen-deficiency-induced osteodecoma. *In vitro* culture of isolated osteoclast precursors from BALB/cJ P2X7R^−/−^ and BaLB vc J P2/cR^+/+^ mice revealed that these precursor types produced a slight increase in the number of mature resorbing osteoblasts, but that each bone type’s ability to absorb bone was significantly reduced. Additionally, osteoblast activity increased in the lack of P2X7R under modified culture conditions, suggesting that the hormone properly control the length of life and activity of bone cells. Additionally, it was shown that even in the absence of P2X7R, bone loss brought on by an elevated estrogen deficiency could be reduced by using mechanical loading as a bone formation stimulus (Wang et al., 2018). Systemic glucocorticoid therapy is the main cause of secondary osteoporosis, which is often brought on by inflammatory autoimmune diseases. Recent studies have shown that the P2Y13R regulates ivory remodeling and guards mice against estrogen deficiency-induced bone loss. Additionally, the P2Y13R acts as a negative feedback tract, preventing human red blood cells from releasing ATP when the oxygen level is low (Boeynaems et al., 2016). These results suggest that the P2Y13R plays a part in the metabolism of ATP and that it may be able to respond to electrical loading through other purinergic receptors, such as those found in P2X7R. As a result, these results suggest potential therapeutic approaches for treating fracture linked to ATP-induced cell death.

### 5.3 ATP-induced cell death and postmenopausal osteoporosis

Postmenopausal osteoporosis (PMOP) is a systemic metabolic bone disorder brought on by low estrogen levels and low bone density in women. Due to increased inflammatory cytokines, particularly TNF, estrogen deficiency causes quick declines in bone volume, density, and strength. Notable studies have shown that P2R is closely related to inflammation, the immune system’s ability to respond to cellular signals, as well as energy metabolism. In particular, the differentiation and proliferation of bone cells depend heavily on the P2X5 (P2X7), P2Y1 (1, and P2/Y2Rs). While mediating inflammation-induced bone damage, the P2X5R controls osteoclast-mediated bone resorption. The P2X2/3R serves two purposes in bone: it mediates bone pain and regulates bone resorption. Additionally, decreased P2X7R function worsens estrogen-depletion-induced bone loss (Fuller et al., 2007; Sekar et al., 2018; Jørgensen, 2019). Additionally, a number of purinergic receptors have been connected to magnesium channels and are closely related to OB, osteoclast, and their progenitors’ distinction and proliferation (Muscella et al., 2018).

The AKT signaling pathway is activated by TNF, which promotes the differentiation of mouse bone marrow hematopoietic stem cells (BMHSCs) into osteoclasts, according to study findings. The homeostasis between OBs and osteoclasts is disrupted, osteoporosis progression is accelerated, and BMHSC P2X7R expression is considerably increased as a result of this process. As a result, inhibiting the P2X7R can significantly lessen TNF-’s effects on BMHSCs and inhibit the formation of osteoclasts (Lu et al., 2020). Additionally, the P2Y2R acts as a detrimental controller of osteoblast differentiation and mineralization, and TNF-upregulates its expression, which contributes to the tumor-differentiation inhibitory effect (Du et al., 2018). According to extra research, low ATP concentrations activate a number of P2XRs (purinergic 2X receptors) in addition to P2, which increases bone resorption, while high and low levels of calcium decrease the capacity of primary rat cells to absorb bone. Importantly, cultured in a lower pH environment significantly improves this stimulating effect on resorption (Morrison et al., 1998). Additionally, P2X5R has been shown to increase osteoblast proliferation when ATP is present (Nakamura et al., 2000). These results suggest a potential therapeutic approach to treating ATP-induced cell death.

## 6 Crosstalk between ATP-induced cell death and other cell death modality pathway components

Recent studies have questioned the uniqueness of ATP-induced cell death, which was once thought to be distinct from other types of suicide. Emerging evidence points to the possibility that ATP-induced mobile death-related factors may also be involved in pyroptosis and autophagy, two other cell-death mechanisms. The discovery of this crosstalk contributes to our understanding of the pathogenesis of osteoporosis and makes it easier to create therapeutic interventions.

### 6.1 Crosstalk between components of cell death and apoptosis induced by ATP

Apoptosis is a highly precise pathway of cell death that is triggered by a variety of signals from both outside and inside the cell, often involving the activation of effector caspases. To regulate this process, the permeabilization of mitochondria plays a crucial role by releasing important pro-apoptotic regulators into the cytoplasm. Within this elaborate regulatory network, the BCL-2 (B cell lymphoma-2) family members orchestrate intricate interactions among three different groups: anti-apoptotic subgroups (such as BCL-2) and pro-apoptotic subgroups [including BAX (BCL-2 Associated X Protein), BAK (BCL-2 antagonist killer), and BH3 domains]. Interestingly, components of these BCL-2 subgroups are also found in the endoplasmic reticulum (ER), an organelle whose emerging significance in apoptosis is just beginning to be understood. In addition to transmitting stress signals that ultimately lead to cell death, the ER plays crucial roles in Fas-induced apoptosis and p53-associated pathways linked to DNA damage and oncogene expression. Within the ER, calcium reservoirs trigger cytoplasmic apoptotic pathways and increase the vulnerability of mitochondria to direct pro-apoptotic stimuli (Breckenridge et al., 2003).

**FIGURE 3:**
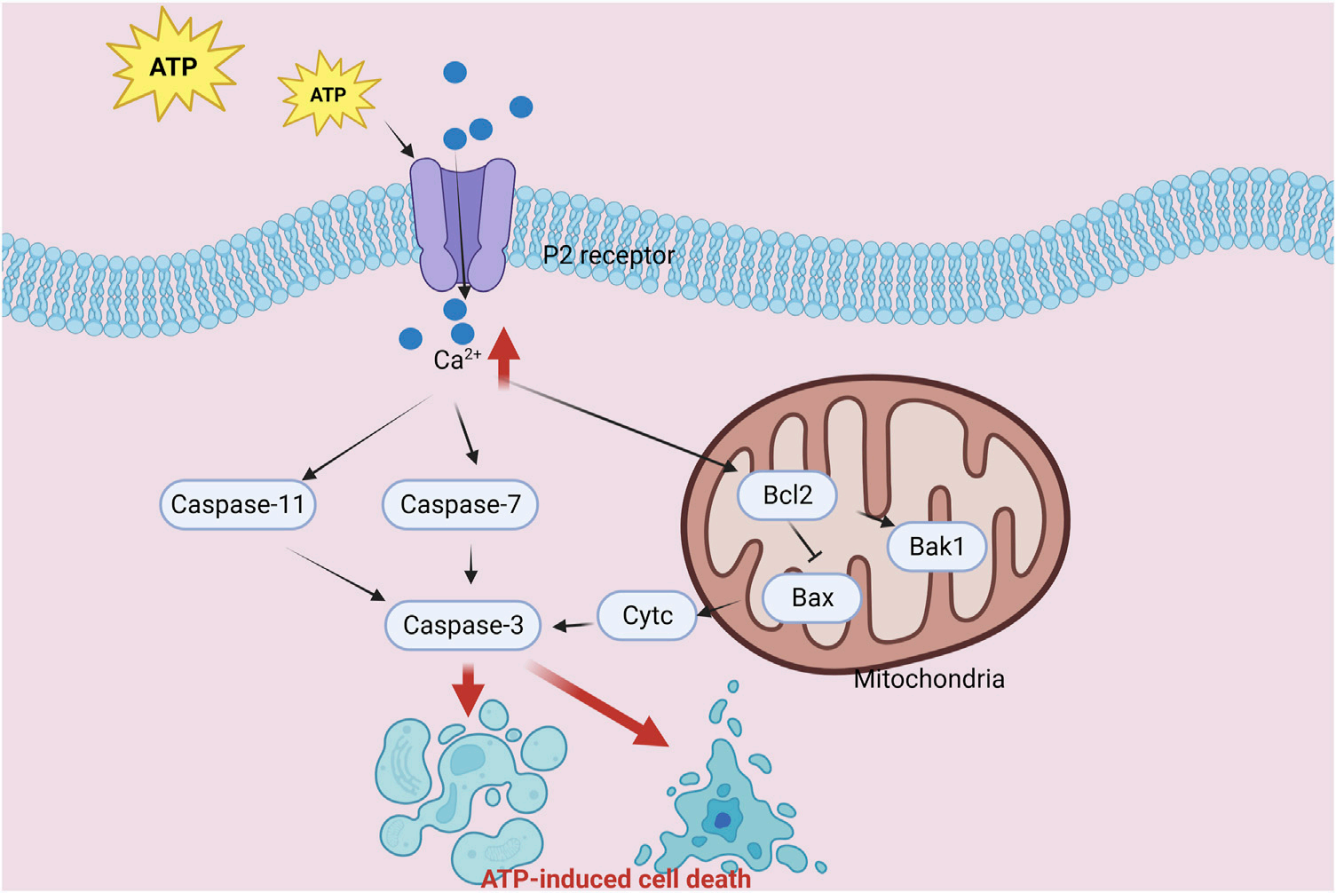
Diagram of the crosstalk mechanism between ATP-induced cell death and apoptosis components.

Extracellular ATP, a potent signaling molecule, regulates various cellular processes by activating P2 purinergic receptors. At elevated extracellular levels, ATP exhibits cytotoxic and inhibitory roles in different cellular systems. A particular study unveiled that ATP stimulation resulted in the suppression of the pro-apoptotic Bax gene, concurrently with reduced Bax protein expression (Figure 3). Additionally, ATP treatment triggered the activation of pro-caspase-3 and the cleavage of poly (ADP-ribose) polymerase (PARP). Importantly, ATP-induced molecular changes in (human embryonic kidney) HEK-P2X7 cells encompassed decreased Bax expression and enhanced PARP cleavage (Wen et al., 2003).

Motor neurons cultured from embryonic rat spinal cords express noticeable levels of P2X7, indicating their vulnerability to caspase-dependent apoptosis when exposed to extremely low concentrations of the P2X7 agonist 2′ (3′)-O-(4-benzoylbenzoyl)-ATP. Interestingly, the natural P2X7 agonist ATP induces death in motor neurons even at low concentrations (1–100 M). Surprisingly, a high concentration of ATP (1 mM) actually provides a protective effect, likely due to the presence of adenosine in the surrounding medium, which activates adenosine receptors. The demise of motor neurons triggered by P2X7 relies on the production of peroxynitrite mediated by neuronal nitric oxide synthase, activation of p38, and autocrine FAS (Facility Associated Signaling) signaling (Gandelman et al., 2013). Taken together, these findings emphasize the participation of ATP-induced pro-apoptotic effects, which are characterized by reduced Bax gene expression and enhanced PARP cleavage. Additionally, the activation of P2X7 in motor neurons triggers apoptosis, involving specific regulatory mechanisms such as the generation of peroxynitrite through the mediation of nitric oxide synthase, the activation of p38, and the autocrine signaling of FAS.

Stimulation of P2X7R by extracellular ATP initiates a series of events, including entry of Na^+^ and Ca^2+^ ions, excessive Ca^2+^ buildup in the cytosol, and subsequent cell death. Interestingly, recent findings indicate that P2X7R activation not only causes Ca^2+^ overload but also triggers Ca^2+^ release through IP3R (inositol triphosphate 3 receptor), continuous depletion of Ca^2+^ reserves, endoplasmic reticulum stress, and eventual programmed cell death (Chao et al., 2012). These groundbreaking discoveries show that apoptosis and ATP-induced cell death are mutually regulated processes. In instance, in postmenopausal osteoporosis, a disorder marked by decreasing bone density, extensive research has demonstrated the critical significance of P2Rs. In human OBs, P2R activation can cause ATP-induced cell death and apoptosis, impairing their functionality. These data taken together clearly imply that a major mechanism underpinning the pathophysiology of osteoporosis is the interaction between apoptosis and ATP-induced cell death. Therefore, successful therapeutic approaches in the therapy of osteoporosis show promise when focusing on the critical components that ATP-induced cell death and apoptosis have in common.

### 6.2 Crosstalk between ATP-induced cell death and autophagy components

During crucial developmental phases, the complex self-degradation process known as autophagy provides a crucial mechanism for preserving energy balance and coping with dietary stress. Additionally, autophagy is essential for the removal of intracellular pathogens as well as the clearing of misfolded proteins, protein aggregates, damaged mitochondria, endoplasmic reticulum, and peroxisomes. The fusing of autophagosomes and lysosomes is a crucial step in this intricate process, which is then followed by destruction carried out by lysosomal proteases. Autophagy is extensively regulated by intracellular signaling networks, including stress-activated kinases (Glick et al., 2010).

After a brain injury, damaged cells produce ATP, which causes microglia to become activated. Tumor necrosis factor (TNF) and other bioactive chemicals are then released by these activated microglia. Notably, the P2X7R appears to be necessary for the release of TNF. Anthra[1,9-cd]pyrazol-6(2H)-one (SP600125), 1,4-Diamino-2,3-dicyano-1,4-bis[2-amino-phenylthio]butadiene (U0126), and 4-(2-) are examples of inhibitors used in pharmacological inhibition investigations. ATP-stimulated TNF generation in microglia has been successfully suppressed by the drug 4-fluorophenyl)-2-(4-methylsulfonyl phenyl)-5-(4-pyridyl) IH-imidazole (SB203580), which specifically targets MEK (mitogen-activated protein kinase kinase), JNK, and p38. Notably, TNF mRNA (messenger ribonucleic acid) production was significantly inhibited by U0126 and SP600125. These results imply a role for ERK and JNK in the control of TNF mRNA expression, p38 in the transport of TNF mRNA into the cytoplasm, and a potential function for PTK, a protein tyrosine kinase belonging to the src family, operating downstream of P2X7R to activate JNK and p38 signaling pathways.

**FIGURE 4:**
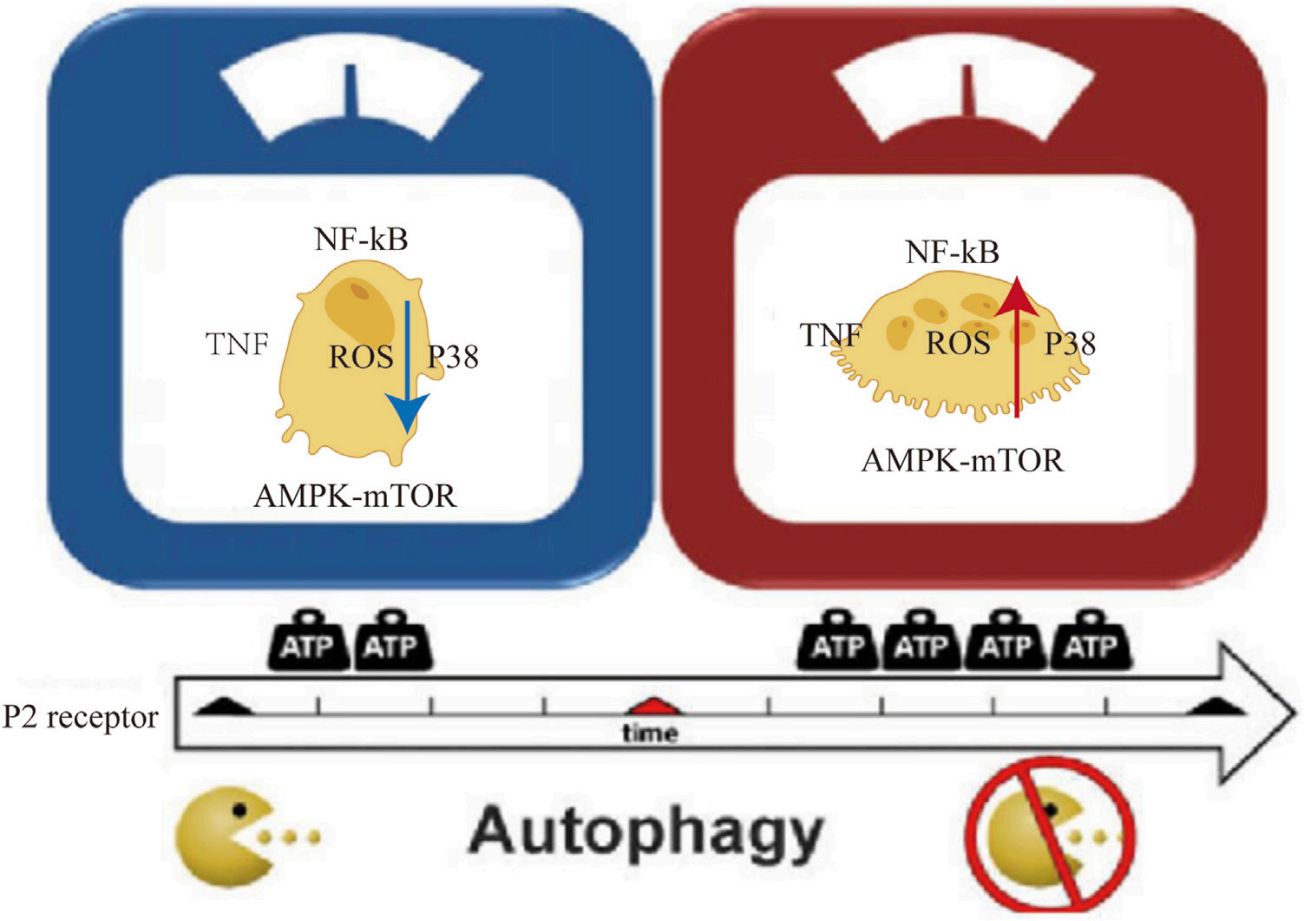
Diagram of the crosstalk between ATP-induced cell death and autophagy components: P2 cell membrane receptor activation, p38 AMPK-mTOR/NF-kB signaling pathway, TNF factor, and intracellular ROS increase with an increase in extracellular oxygen (ATP).

mTOR (mammalian target of rapamycin) kinase is the key regulator of cellular autophagy levels. It is a key signaling molecule that works by inhibiting the ATG1/Ulk-1/-2 (antibodies to autophagy associated protein 1) complex during the early stages of lipid bilayer phagophore formation. The integration of metabolic, growth factor, and energy cues depends heavily on mTOR. When nutrients are abundant, mTOR suppresses autophagy while enhancing growth-related processes such protein translation that are induced by mTOR signaling (Glick et al., 2010). The beginning and completion of autophagy are closely regulated by the ATP-induced cell death process. Increased ATP levels trigger the AMPK (5′ AMP-activated protein kinase) and mTOR signaling pathways, both of which are essential for controlling autophagy. Initiation of autophagy is encouraged by AMPK activation, whereas autophagy is enhanced by mTOR suppression (Figure 4). The primary regulatory point for transferring extracellular ATP signals is P2X7. Our research has shown that the P2X7-AMPK-PRAS40-mTOR axis, which was recently found, and the well-known P2X7-PI3K/AKT axis make up the two pathways that make up the downstream intracellular signaling network. These two mechanisms upset the equilibrium between growth and autophagy when exposed to high extracellular ATP levels, hence encouraging tumor cell death (Zhang et al., 2019). Contrarily, growth factor signaling mediated by the insulin receptor and its adaptor IRS1, as well as other growth factor receptors activating Akt and Class IPI3 kinase, inhibits autophagy, boosts mTOR activity, and increases Rheb GTPase (Ras homolog enriched in brain) activity by inhibiting TSC1/TSC2 (tuberous sclerosis complex gene 1/2) activity (Glick et al., 2010). Autophagy and ATP-induced cell death have complex linkages and crosstalk that are regulated by the same regulatory mechanisms and signaling pathways that control cell destiny and environment adaptation. Our comprehension of their interrelationship will be improved by further research, which will also present fresh approaches and targets for the treatment of diseases that are connected.

### 6.3 Crosstalk between ATP induced cell death and pyrogenic components

Pyroptosis, a type of programmed cell death closely related to inflammation, is characterized by cell lysis, swelling, and the release of pro-inflammatory cytokines (IL-18 and IL-1) (Ji et al., 2021). The linker region connecting the C- and N-terminal domains of GSDMD, which allows the insertion of M. g. N into the cell membrane lipid membrane and the formation of pores via its N, is then cleaved after being activated, such as in the case of caspase-1. Osmotic cell lysis is induced by this process, which results in pyrodeath (Feng et al., 2018; Ru et al., 2023). Two main pathways-the caspase-3/GSDME pathway and the GSDMD pathway, which is controlled by casese-1/4/5/11-are responsible for the initiation of pyrotosis (Ru et al., 2023).

**FIGURE 5:**
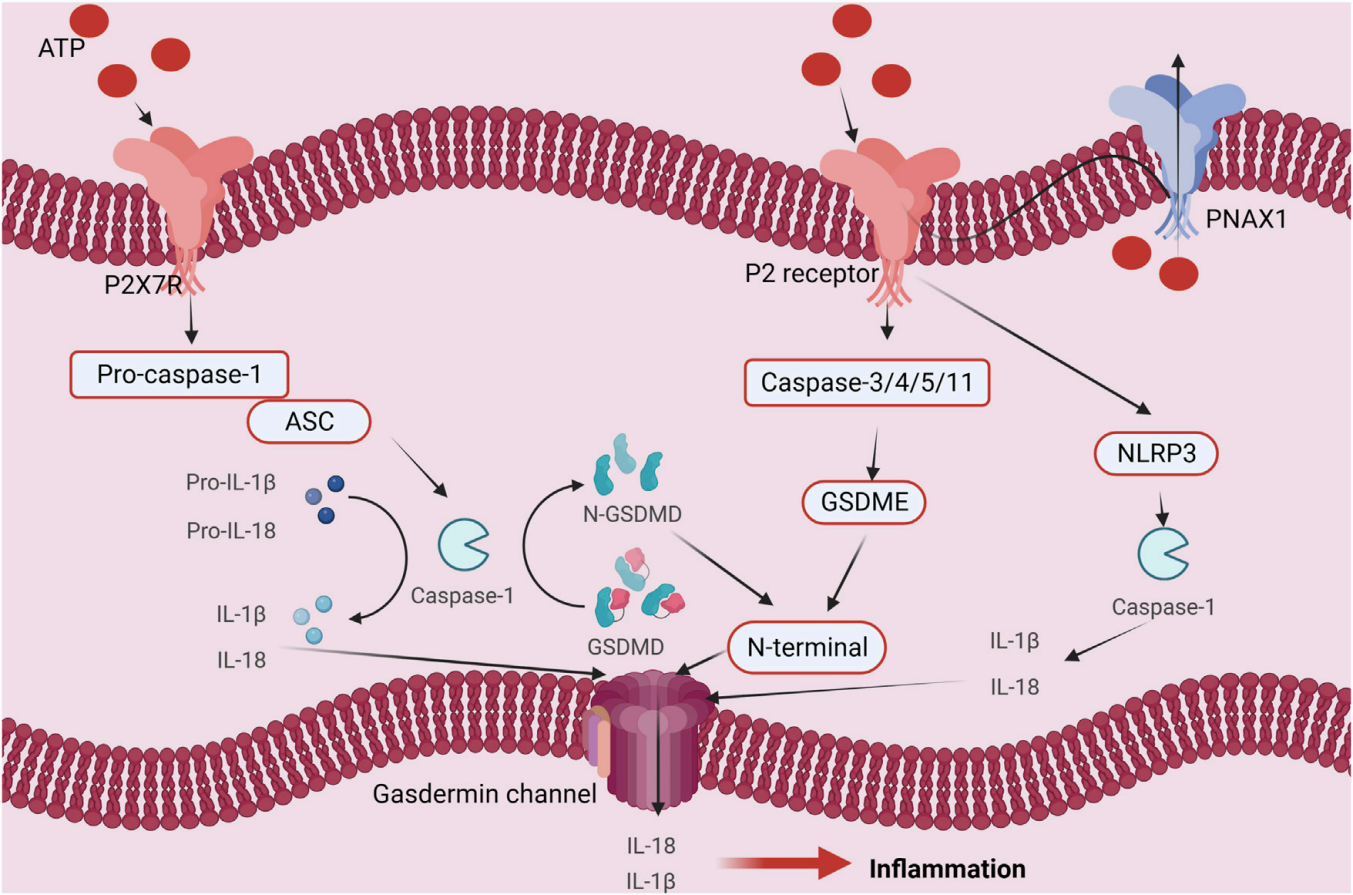
Crosstalk mechanism between ATP-induced cell death and pyroptosis components.

The P2X7R’s extracellular ATP activation opens cation channels/pores, allowing for significant efflux of K^+^ as well as associated processing and secretion of the pro-inflammatory cytokines IL-1 and 18(IL-18). The release of microvesicles/exosomes, membrane foaming, and the shedding of specific surface molecules are just a few minutes after P2X7R activation (Wiley et al., 2011). ATP, a traditional activator, can cause macrophages to activate NLRP3 inflammasomely, leading to caspase-1/GSDMD-mediated pyrodeath. According to one study, ATP treatment did not cause caspase-1 activation or GSDMD cleavage but did cause cell lysis death that anatomically resembled common pyroptosis. Again, apoptotic delegates (caspase-8 and caspase-9) were significantly actuated, along with apoptotic effectors (caspase-3 and caspase-7), performing in thefractionalization of GSDME toinduce its N-terminalscrap (GSDME-NT) andprosecution of cell pyrosis. Inhibition of caspase- 3 reduced ATP-convinced GSDME-NTproduct and lytic cell death (Zeng et al., 2019b) (Figure 5). Specially, cell death and pyrodeath are intricate processestoldby multiple factors and nonsupervisory pathways. Cell typeparticularity, pathological conditions, and microenvironmental factors may modulate the relationship between ATP-convinced cell death and necrosis. Fartherexploration isneeded to comprehend the interplay and nonsupervisory mechanisms governing thesemarvels.

### 6.4 Crosstalk between ATP induced cell death and ferroptosis

The quintessential process of ferroptosis involves lipid peroxidation mediated by bivalent iron or esterase oxygenases that catalyze the proliferation of unsaturated fatty acids on cellular membranes. Concurrently, this process is characterized by a diminution of GPX4 (glutathione peroxidase 4), the quintessential enzyme governing the antioxidant system (glutathione system) (Gao et al., 2022; Jiang et al., 2022). In iron-abundant milieus, extracellular ATP augments iron assimilation and potentiates mitochondrial ATP synthesis. The ATP-Fe complex precipitates iron-centric apoptosis in susceptible cells through a synergy of ATP and Fe^2+^. Moreover, energy stress can modulate mitochondrial ATP dynamics, either fortifying or mitigating AMPK regulation of iron homeostasis (Tian et al., 2023). Intriguingly, P2RX7 antagonism mitigates the deleterious impacts of high glucose environments, markedly adjusting the expression of pivotal proteins like P53 (tumor protein p53), HO-1 (heme oxygenase 1), and p-ERK1/2 while elevating SLC7A11 and GPX4 levels. This suggests that curbing the ATP-induced P2RX7 receptor diminishes oxidative duress and iron accrual in endothelial cells, both *in vivo* and *in vitro* (Liu et al., 2023). The AMP-activated protein kinase (AMPK), a pivotal sentinel and arbiter of cellular energetic equilibrium, responds to intracellular AMP-ATP ratios and is actuated under AMP ascendancy. Once activated, AMPK orchestrates an intricate cascade that mitigates ATP depletion and bolsters synthesis, thereby reestablishing AMP-ATP balance and ensuring the sustenance of cellular physiological functions. Emerging research posits that AMPK activation engenders Nrf2 (Nuclear factor erythroid 2-related factor 2) activation and nuclear translocation, fortifying cellular antioxidant capacities and impeding ferroptosis (Chen et al., 2023). These findings denote a potential entanglement or confluence of mechanisms between ATP-induced cell demise and ferroptosis, warranting further elucidation.

## 7 Conclusion and outlook

In the context of osteoporosis, this thorough review clarifies novel regulatory mechanisms underlying ATP-induced cell death. ATP has a significant impact on the pathogenesis of osteoporosis because it is an important intracellular signaling molecule. ATP causes bone cell death and influences the progression of osteoporosis by modulating cell membrane surface receptors, immune inflammation, and mitochondria-related signaling pathways. Extensive research emphasizes the importance of ATP-induced cell death in osteoporosis and its complications, pointing to the possibility that treatments for patients with the condition could provide promising therapeutic options.

However, a number of difficult issues call for this field’s attention. First off, it is still difficult to precisely identify the various types of ATP-induced cell death. A deeper comprehension of osteoporosis’ underlying mechanisms will be made possible by distinguishing between apoptosis, necross, autophagy, and other types of cell death and determining their relative contributions. Second, there are not enough specific biomarkers to recognize ATP-induced cell death in a clinical setting. The serum of osteoporosis patients and its associated complications contains altered levels of some ATP-related proteins, such as P2Rs, but their specificity currently does not meet the needs of clinical diagnostics. The specific mechanisms governing ATP-induced cell death in osteoporosis will be revealed by further investigation of molecular mechanisms, including those encompassing interactions between the two receptors, the regulation of signal transduction pathways, and changes in the level of active oxygen (ATP) in both internal and external cells. In order to slow the onset and development of osteoporosis, therapeutic approaches must also be developed, such as focusing on ATP amounts, interfering with pertinent receptors or signaling pathways, or creating new targets. Finally, intensified medical research and translational medicine efforts are required to translate laboratory research into clinical applications, which include the creation of diagnostic, preventive, and treatment strategies. In order to further our understanding and treatment of osteoporosis, potential research projects will address these issues while incorporating translational medicine strategies. Targeting only one cell death pathway, such as apoptosis, autophagy, necrosis-, or ferropto-, may never produce the desired healing results in cases where multiple types of programmed cell mortality are involved in osteoporose complications. Therefore, in order to effectively address the complexity of this disease, potential treatment strategies may need to adopt multi-target therapeutic approaches.

We outline a strategic approach for the validation of ATP-induced cell death within a rigorous experimental framework. Rats were meticulously chosen as experimental animals, meticulously allocated into distinct groups - one being the experimental cohort and the other serving as the control counterpart. The experimental group received precisely calibrated doses of ATP, administered either intravenously or intraperitoneally, while the control group was subjected to a saline solution or an equivalent under identical conditions. Rigorous monitoring of physiological parameters, meticulous collection of tissue samples, discerning the markers indicative of cell demise and inflammatory responses, and in-depth evaluation of molecular mechanisms ensued. To scrutinize the potential impact, drug interventions were judiciously implemented. Further assessment involved the scrutiny of mitochondrial functionality in tissues, encompassing factors such as mitochondrial membrane potential and ATP synthesis levels. The experimental group underwent exposure to ATP receptor antagonists and mitochondrial protectors to ascertain the impact of these pharmaceuticals on ATP-induced cell death. Following stringent statistical analyses, comprehensive insights were gleaned to elucidate whether ATP indeed prompted cell death in the animal subjects and the intricacies of its underlying mechanism.
